# Author Correction: Role of astroglia in Down’s syndrome revealed by patient-derived human-induced pluripotent stem cells

**DOI:** 10.1038/s41467-020-14865-1

**Published:** 2020-02-21

**Authors:** Chen Chen, Peng Jiang, Haipeng Xue, Suzanne E. Peterson, Ha T. Tran, Anna E. McCann, Mana M. Parast, Shenglan Li, David E. Pleasure, Louise C. Laurent, Jeanne F. Loring, Ying Liu, Wenbin Deng

**Affiliations:** 1 Department of Biochemistry and Molecular Medicine, School of Medicine, University of California, Davis, California 95817 USA; 2 Institute for Pediatric Regenerative Medicine, Shriners Hospitals for Children, Sacramento, California 95817 USA; 3 Department of Neurology, Institute of Neurology, Tianjin General Hospital, Tianjin Medical University, Tianjin, 300070 China; 4 Department of Neurosurgery, University of Texas Health Science Center at Houston, Houston, Texas 77030 USA; 5 Center for Stem Cell and Regenerative Medicine, The Brown Foundation Institute of Molecular Medicine for the Prevention of Human Diseases, University of Texas Health Science Center at Houston, Houston, Texas 77030 USA; 6 Department of Reproductive Medicine, University of California, San Diego, La Jolla, California 92037 USA; 7 Center for Regenerative Medicine, Department of Chemical Physiology, The Scripps Research Institute, La Jolla, California 92037 USA; 8 Department of Pathology, University of California, San Diego, La Jolla, California 92093 USA; 9 Present Address: Department of Biology, University of Washington, Seattle, Washington, 98195 USA

Correction to: *Nature Communications* 10.1038/ncomms5430, published online 18 July 2014.

The original version of this Article omitted the following from the Acknowledgements:

L.C.L. and M.M.P. were supported by CIRM (RN2-00931).

This has not been corrected in the PDF and HTML versions of the Article.

